# An observational study on quality of life and preferences to sustain life in locked-in state

**DOI:** 10.1212/WNL.0000000000008064

**Published:** 2019-09-03

**Authors:** Magdalena Kuzma-Kozakiewicz, Peter M. Andersen, Katarzyna Ciecwierska, Cynthia Vázquez, Olga Helczyk, Markus Loose, Ingo Uttner, Albert C. Ludolph, Dorothée Lulé

**Affiliations:** From the Department of Neurology (M.K.-K., K.C.) and Neurodegenerative Diseases Research Group (M.K.-K.), Medical University of Warsaw, Poland; Institute of Pharmacology and Clinical Neuroscience (P.M.A.), Umeå University, Sweden; and Department of Neurology (C.V., O.H., M.L., I.U., A.C.L., D.L.), University of Ulm, Germany.

## Abstract

**Objective:**

This is an observational study on well-being and end-of-life preferences in patients with amyotrophic lateral sclerosis (ALS) in the locked-in state (LIS) in a Polish sample within the EU Joint Programme–Neurodegenerative Disease Research study NEEDSinALS (NEEDSinALS.com).

**Methods:**

In this cross-sectional study, patients with ALS in LIS (n = 19) were interviewed on well-being (quality of life, depression) as a measure of psychosocial adaptation, coping mechanisms, and preferences towards life-sustaining treatments (ventilation, percutaneous endoscopic gastroscopy) and hastened death. Also, clinical data were recorded (ALS Functional Rating Scale–revised version). Standardized questionnaires (Anamnestic Comparative Self-Assessment [ACSA], Schedule for the Evaluation of Individual Quality of Life-Direct Weighting (SEIQoL-DW), ALS Depression Inventory–12 items [ADI-12], schedule of attitudes toward hastened death [SAHD], Motor Neuron Disease Coping Scale) were used, which were digitally transcribed; answers were provided via eye-tracking control. In addition, caregivers were asked to judge patients' well-being.

**Results:**

The majority of patients had an ACSA score >0 and a SEIQoL score >50% (indicating positive quality of life) and ADI-12 <29 (indicating no clinically relevant depression). Physical function did not reflect subjective well-being; even more, those with no residual physical function had a positive well-being. All patients would again choose the life-sustaining techniques they currently used and their wish for hastened death was low (SAHD <10). Caregivers significantly underestimated patient's well-being.

**Interpretation:**

Some patients with ALS in LIS maintain a high sense of well-being despite severe physical restrictions. They are content with their life-sustaining treatments and have a strong will to live, which both may be underestimated by their families and public opinion.

The locked-in state (LIS) is a rare phenomenon, which may occur in various diseases and is characterized by complete immobility and loss of verbal communication abilities (anarthria) while being fully conscious (deefferentiation).^1^ In classic LIS, communication with the outer world is only possible using eye movements, while in residual LIS some additional motor abilities, such as movement of the toes, might be available. LIS may also occur in amyotrophic lateral sclerosis (ALS), which is pathologically characterized by progressive loss of efferent control leading to tetraplegia, anarthria, muscle wasting, respiratory insufficiency, and death within 2 years on average. Patients may survive longer only if therapeutic devices such as noninvasive ventilation (NIV)^2^ or invasive ventilation and percutaneous endoscopic gastrostomy (PEG) are provided.^3^ As most patients in LIS live at home with greatly reduced contact with the outside world, little is known about their thoughts, feelings, and wishes in this condition. For healthy people, LIS may seem among the most threatening conditions, and quality of life (QoL) is generally considered low and depression highly prevalent.^4^ Patients are discouraged to choose life-prolonging treatments such as invasive ventilation as the usage “appears [to be] associated with a lesser quality of life […and a] fear of a locked-in state.” ^5^ On the contrary, previous studies have suggested that loss of physical function is not necessarily associated with low well-being^6^ despite the usage of invasive ventilation.^7^ Shortly after diagnosis, well-being may decline in some patients because of a reactive episode following the diagnosis.^8,9^ If patients readjust their expectations according to the test, operate, test, exit (TOTE) model^10^ and allow for successful coping in the sense of response shift (changing attitudes on what is important in life), reframing (re-evaluating things in life), and reappraisal (looking at things from a different perspective), psychosocial adaptation may occur in the sense of satisfactory QoL and low depression rate^11^ compared to the general healthy population^4^ and despite the use of invasive techniques to support life.^7^ This may not be specific to ALS but may be a general capacity to adjust to adverse circumstances.^10,12^ Little is known about patients with ALS’ satisfaction with life-sustaining treatments or their wish to end life in the context of their well-being and coping strategies. Previous research has provided evidence that patients in LIS may have a strong will to live,^12,13^ but quality is limited by the fact that most studies so far used simple blink Morse code to communicate with the patients or focused primarily on well-being. We present a unique explorative approach of communication with patients in LIS via eye-tracking control to learn not only about their well-being,^11^ but also about their therapeutic decision-making and end-of-life preferences in a state of complete physical restriction (for further details, see NEEDSinALS.com).

## Methods

### Participants

**Figure 1:**
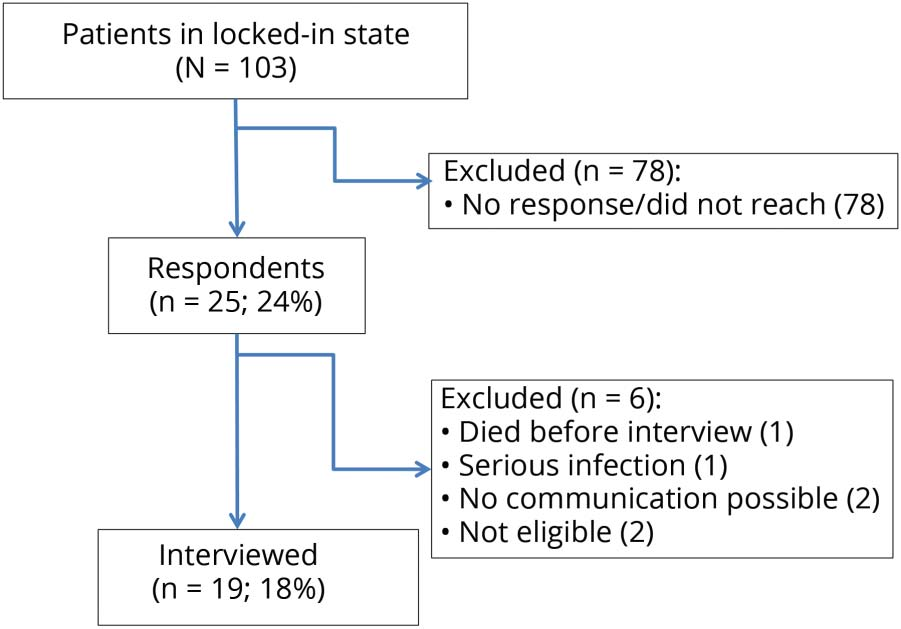
Flowchart of patient recruitment Patients and caregivers were invited to the study in 2 steps: (1) there was a general announcement on the patient organization's webpage and Facebook account; (2) there were personal emails sent to 103 patients or caregivers (depending on the provided emails on registration to the association; all had applied previously for financial aid, which is exclusively granted for patients with amyotrophic lateral sclerosis in advanced stages). The personal emails enclosed the questionnaires in addition to documents provided by local neurologists/general practitioners that included information on the use of percutaneous endoscopic gastrostomy (PEG), noninvasive ventilation (NIV)/invasive ventilation, and the result of the Barthel scale. Only patients with PEG, NIV/invasive ventilation, and Barthel scale 0–20 (10 given for controlling bladder and bowel functions, each) were considered potential patients in locked-in state and were contacted by the study team. Additional help was offered by 2 respiratory team nurses, who actively informed patients who were under their care, their doctors, and their caregivers about the study.

In this exploratory cross-sectional study, 103 caregivers of patients in the LIS were contacted by email by Dignitas Dolentium, the Polish patient organization. Caregivers of 25 patients responded (24%; 78 were not reached or did not respond; all those who were contacted had PEG, invasive ventilation/NIV, and Barthel scale 0–20, so there was no clinical difference between those who responded and those who did not) and 19 (18% of all registered patients in LIS in the Polish patient organization) were interviewed between December 2016 and January 2017 (table); 1 patient died before the interview, 1 developed a serious infection, with 2 no communication was possible, and 2 were not eligible (figure 1). There were both active patients and active caregivers who encouraged their proxies to participate. We visited and interviewed patients at their homes throughout Poland. Inclusion criteria were clinical diagnosis of ALS^14^ with classical LIS with severe physical restriction but (almost) complete loss of purposeful volitional motor control for communication (either verbally or written) and walking. Exclusion criteria were an overt cognitive impairment certified by a trained neuropsychologist (M.L. and O.H.) or clinically defined frontotemporal dementia according to Rascovsky criteria.^15,16^

**Table:**
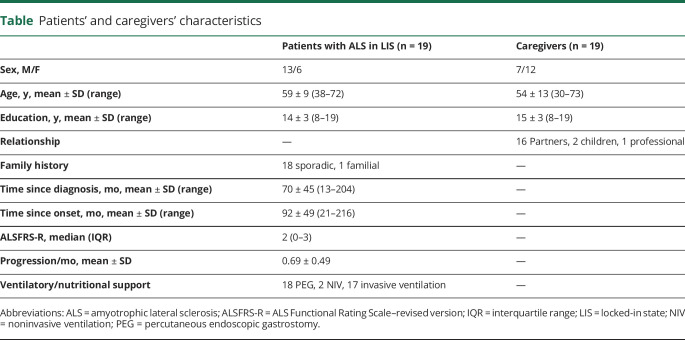
Patients' and caregivers' characteristics

All patients had used NIV to start with, but at the time of the study, most patients (17/19) used invasive ventilation via tracheostomy and PEG. Two patients had declined to use invasive ventilation and continued to use NIV via facial mask. In total, 12 patients (71%) received invasive ventilation in an emergency situation, including 7 with no prior information on the measures, 5 with a prior positive attitude. Those five patients with a prior positive attitude towards invasive ventilation underwent a planned tracheostomy. In Poland, there are no legal means to discontinue invasive ventilation. There was no clinical difference between those who had planned invasive ventilation/PEG and those who did not with regard to time since onset/diagnosis, ALS Functional Rating Scale - revised version (ALSFRS-R), age, or years of education (all *U* < 1.31 with *p* > 0.05).

### Peer assessment

In addition, patients' caregivers were asked to judge the patients' well-being. Most of them were partners or other close family members; only one was a professional carer. Caregivers lived at the patient's home and were mostly female (table).

### Standard protocol approvals, registrations, and patient consents

The study was approved by the Ethics Committee of the University of Ulm (19/12) and Warsaw (KB/138/2013). All participants provided written (for patients via eye-tracking) informed consent.

### Means of communication

**Figure 2:**
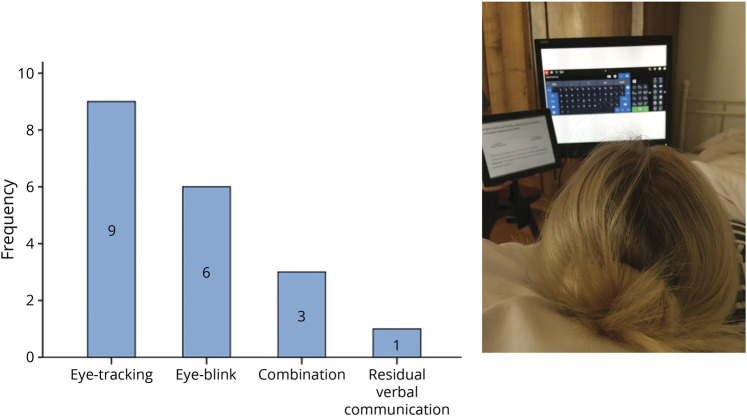
Experimental setup Mode (left) and setup (right) of the interviews in 19 patients in locked-in state. The Tobii eye-tracker was placed below the screen, on which the virtual keyboard was presented. Patients fixated targets consecutively (letters or numbers); the eye-tracker recognized the gaze direction and recorded the response above the virtual keyboard so that patients could see what they had typed (the white field on the table screen on the right image).

Patients provided their answers either via eye-tracking control on a visual keyboard or via blink responses or via both. Questionnaires were presented on a portable screen (tablet PC) and in addition, the interviewer asked the question. Therefore, the patients had both verbal and auditory input. For those 12 patients who used the eye-tracker system, a virtual keyboard was presented on a tablet screen. This tablet was placed about 30 cm in front of the patient and a mobile telescope arm (possible rotation 360°) was used for fixation of the tablet so patients could either sit or lie in bed. Patients fixated targets consecutively (letters or numbers) with their eyes and thus “typed” their answers letter by letter. Gaze was tracked by a standard eye-tracking device with infrared light (Tobii Technologies, Stockholm, Sweden) placed below the laptop screen or by an individual device owned by the patient (figure 2). Responses were presented above the virtual keyboard so patients were able to see what they had typed. Self-corrections were possible by using the “delete” button on the virtual keyboard.

Questions and choices for answers were visually and verbally presented by the interviewer and 9 patients indicated their choices via eye-tracking, 6 via eye-blink, 3 patients used a combination of both, and 1 patient had residual verbal communication skills (but was completely tetraplegic). The interview lasted between 3 and 4 hours with breaks in-between to allow the patient some rest ad libitum*.*

On a daily basis, only 1 patient used eye-tracking technique for communication; all other patients had no access and used blinking with the eyes to indicate responses to others (blink Morse code). One patient had previously used the Tobii eye-tracking system and one patient a sensor system by foot control, but no techniques were used at the time of the study.

### Measures

#### Clinical data

To reduce patient load, clinical data were provided by caregivers where possible. Physical function was measured with the ALS Functional Rating Scale–revised version (ALSFRS-R) with a range of 0–48 where 0 defines no movement abilities of the extremities and complete anarthria.^17^ Progression was determined as total loss on the ALSFRS-R divided by the time since diagnosis in months (48 − current ALSFRS-R/months since diagnosis). None of the patients presented with severe cognitive or behavioural deficits certified by the trained psychologists O.H. and M.L. Minor cognitive deficits can be assumed to not interfere with well-being or medical decision-making.^18^

#### Psychosocial adaptation

Measures of psychosocial adaptation were determined with standardized questionnaires. Patients were asked to evaluate their QoL and affective state (depressiveness) as measures of emotional and psychological adjustment to the condition. Global QoL was determined using the Anamnestic Comparative Self-Assessment (ACSA),^19^ where the patient is asked to judge his or her global quality of life in relation to the worst (−5 on Likert scale) and best (+5) experience in one's own life (≥0 indicates positive QoL). Furthermore, subjective QoL was determined with the Schedule for the Evaluation of Individual Quality of Life-Direct Weighting (SEIQoL-DW),^20^ where patients determine their 5 most relevant fields of QoL, the share of each field for the subjective QoL, and the overall satisfaction with this field. A SEIQoL index score can be determined (range 0%–100%; ≥50 indicates satisfactory QoL).

Depressiveness was determined with the ALS Depression Inventory–12 items (ADI-12),^21^ which addresses the patient's affective state. Response options are fully agree (1) to fully disagree (4), adding up to scores 12–48, where scores >28 are indicative of clinically relevant depression.

Patients indicated wish for hastened death with the schedule for attitudes towards hastened death with binary options (correct/wrong) to respond to 20 statements on wish for hastened death (SAHD; range 0–20),^22^ with a score ≥10 indicating a clinically significant wish.

In addition, patients indicated satisfaction with therapeutic techniques and machines they used for support of breathing (noninvasive and invasive ventilation) and nutrition (PEG) in a semi-structured interview based on previous questionnaires.^8,23^

#### Coping

Patients' coping was determined with the Motor Neuron Disease Coping Scale, encompassing 22 items that can be subsumed under 6 subscales of support, positive action, independence, avoidance, information seeking, and positive thinking.^24^ Responses were provided on a Likert scale from never (1) to always (6).

#### Peer assessment

For caregiver's evaluation of patient's well-being, ACSA and ADI-12 were used with the request to judge the patient's QoL and depressiveness from the caregiver's perspective with the instruction “What do you think would be the patient's response?”

### Statistics

Data were analyzed for normal distribution a priori using the Kolmogorov-Smirnov test and statistics were selected accordingly. Spearman ρ correlation analyses were used to determine correlation coefficients between clinical data (ALSFRS-R, time since disease onset, progression rate determined as current ALSFRS-R score minus 48 divided by the time since disease onset) and well-being (ACSA, SEIQoL, ADI-12; coping strategies). In order to detect group differences between patients with ALS and caregiver responses and between those with planned compared to unplanned therapeutic measures, Mann-Whitney *U* test was used. All analyses were performed using IBM SPSS (Chicago, IL) version 21.0, 2-sided, and the significance level was set at *p* < 0.05. Due to the explorative nature of the study, no correction for multiple comparisons was applied.

### Data availability

Any anonymized data not published within the article will be shared by request from the corresponding author.

## Results

### Well-being of patients in LIS

Patients reported a median global QoL in the ACSA of 1 (Q = −2 to 3); 8 patients reported scores below 0 and the majority of 11 patients reported scores between 1 and +5. Median subjective QoL measured with the SEIQoL was 66 (Q = 58–88), with 17 patients reporting scores between 53 and 98; 2 patients reported a SEIQoL score below 50. Depressiveness measured with the ADI-12 showed a median score of 25 (Q = 22–30); 27% presented no signs of depressiveness, 47% presented with a dysthymic affective state, and 26% presented with clinically relevant depression.

### Association of clinical measures, well-being, and coping

**Figure 3:**
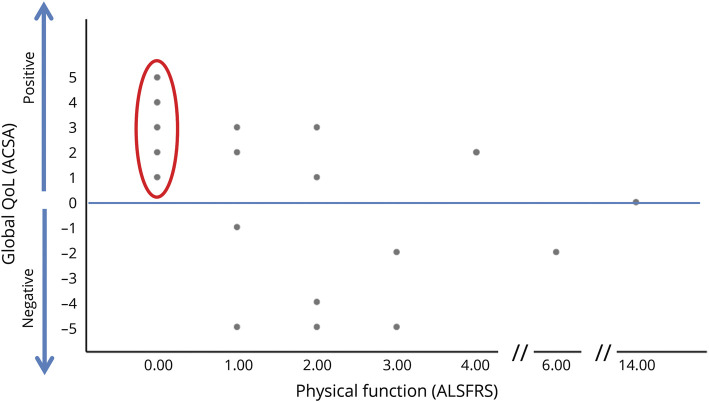
Well-being and physical function loss Association of physical function loss according to ALS Functional Rating Scale - revised version (ALSFRS-R) (range 0–48, where 0 indicates no residual movements and anarthria) and global quality of life (QoL). ACSA = Anamnestic Comparative Self-Assessment.

Physical function did not reflect subjective well-being. We evaluated the association of disease measures and QoL or depression, respectively (low patient numbers and low variance of the ALSFRS-R in LIS does not allow for any reliable statistical analyses). Tentatively, there was no association of QoL (ACSA, SEIQoL) or depression (ADI-12) with time since diagnosis (all *r* < 0.03), progression (all *r* < 0.3), or physical function loss (all *r* < 0.3, apart from association of ACSA and ALSFRS-R with an increasing QoL, the lower the physical function, Spearman ρ correlation with, probably explained by skewed distribution). Even more, those 5 patients (26%) patients with no residual physical function (ALSFRS-R = 0) reported positive QoL and no clinically significant depression (figure 3).

Regarding coping strategies, information-seeking increased with time since diagnosis only (*r* = 0.60), but no other association of coping strategies and clinical measures was found (all *r* < 0.3).

### Patient preferences for medical decision-making

**Figure 4:**
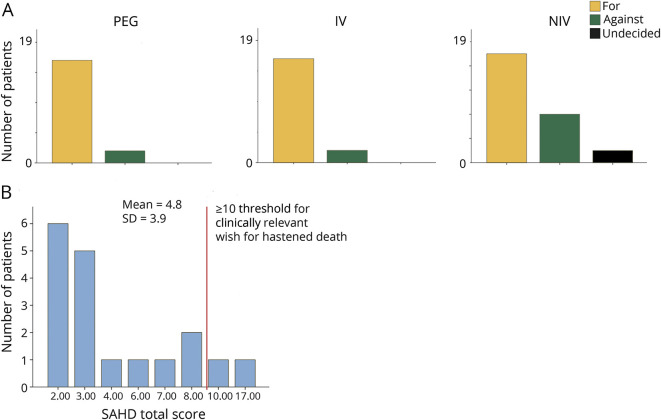
Patients' perspective on life (A) Patients' preferences for therapeutic options if they had to choose again and (B) their wish for hastened death according to schedule of attitudes toward hastened death (SAHD). IV = invasive ventilation; NIV = noninvasive ventilation; PEG = percutaneous endoscopic gastrostomy.

If patients had to choose a second time, most patients would again select invasive ventilation and PEG (17/19). There were only 2 patients on NIV who had decided against invasive techniques of invasive ventilation and PEG (figure 4). Five of the 17 patients in favor of invasive ventilation would directly choose invasive ventilation instead of first using NIV.

### Patient wish for hastened death

Patients presented with a median wish for hastened death of 4.5 (Q = 2 to 7.25); 2 patients had a clinically relevant wish for hastened death (figure 3).

### Differences between patients with planned or unplanned measures

There was no difference in outcome measures between those who had planned invasive ventilation/PEG and those who did not with regard to depressiveness, QoL, coping strategies, or wish for hastened death (all *U* < 4.55 with *p* > 0.05).

### Caregiver evaluation of patient well-being

Caregivers significantly overestimated depressiveness (*U* = 78, *p* = 0.004) and underestimated QoL, but this difference was not significant (*U* = 136, *p* = 0.20).

## Discussion

Due to an overall lack of verbal or written communication, little is known about the psychological state,^11^ coping mechanisms, and therapeutic preferences of patients in LIS. In our study, patients reported a satisfactory QoL and a rate of clinically significant depression of 26% (comparable to patients with ALS in early phases of the disease in Poland, and with an increased rate of depression and dysthymic state compared to other European countries)^23^ despite this devastating condition and dependence on (invasive) therapeutic devices, which supports previous results.^11,12^ In many studies, physical function is regarded as a measure of QoL, which is of course low in patients who are unable to walk, communicate, or undertake any activities. From the perspective of a healthy person, LIS is often among the most threatening scenarios one can imagine and prolongation of life is discouraged due to “fear of a locked-in state.”^5^ Our results indicate, however, that the human being is more than just physical function, and that subjective (hedonic) QoL can be preserved despite physical limitations. Accordingly, the most physically restricted patients in LIS from our study showed a great sense of well-being. As supported by social studies, despite objectively negative factors, these patients found their way to adapt to the condition (“well-being paradox”).^25^ This was even true for patients in LIS in Poland with much lower socioeconomic support than in many other Western countries, where patients with ALS have been shown to have even higher well-being compared to Polish patients.^23^ High religiosity in Poland might be suspected to facilitate and explain the reported positive well-being^26^ and strong will to prolong life. But the contrary has been shown in Polish patients with ALS compared to other European countries with a comparably low QoL and a general preference to decline life-prolonging treatments despite high religiosity.^23^

From previous research, we know that similar psychosocial adaptation may also happen in other fatal disorders (e.g., patients with cancer in palliative treatment)^27^ and requires some preconditions in ALS: it takes time (individuals with a fast disease progression rarely show similarly good results^4^) and strong social support^28^ (like the patients in this study, all of whom had a primary caregiver living in the same household) so the patients in LIS presented here may represent a biased sample. Still, low physical function is not necessarily associated with low well-being, as outlined in previous work.^11,12^ Also, internal resources need to be accessible, which may explain more than 60% of the well-being variance in ALS.^28^ Often, patients with good psychosocial adaptation are also the ones who are well-informed, as shown in the current study: the longer the disease duration, the more information seeking.

Patients in the current study were satisfied with the life-prolonging treatment they used, especially the invasive techniques of invasive ventilation and PEG, although most of them did not prefer these measures in the first place. This supports previous findings that once decisions for these means are taken, patients rarely change their preferences^8,29^; even more, the patients in the current study would volitionally choose them if there was a second choice. Accordingly, these most invasive techniques are the only way to secure life and thus they are cherished by the patients.^2^ In the currently studied group, patients presented with both a high preference for therapeutic devices and a low wish for hastened death, indicating a strong will to live, similar to previous studies of patients with ALS in less advanced stages of the disease.^8^ This might come as a surprise to healthy people, who are rarely confronted with these circumstances. Healthy people without experience with ALS are unable to correctly anticipate this fact^8^ and only experienced health care providers in the field of ALS and palliative care are able to correctly anticipate the emotional state^30^ and the disability paradox^25^ of a patient in LIS. Even the caregivers (mostly spouses) in the current study were unable to correctly anticipate the well-being of their loved ones and were unable to imagine that for the patient the condition was acceptable. We know from previous work that caregivers are physically, mentally, and financially heavily burdened by the circumstances of the disease^31^ and caregivers rather extrapolate from their own feelings so the worse their own mood, the worse they expect the patient's mood to be.^4^ Instead, many patients with ALS are “strong fighters” with a positive attitude despite their desperate physical condition.

Patients in LIS may find a way to readjust their expectations and emotionally adapt to the situation although a majority received invasive life-sustaining treatments in an emergency situation without any means to discontinue.^28^ This capacity to adapt has been reported by other patients in LIS with different etiology and strong social support.^12^ This prerequisite is also a limitation of the current study: the patients interviewed for this study were those who had supportive families (who answered our invitation indicating the patients' willingness to participate). So the presented data might be true for patients who are interested in communicating their messages to the outside world or who have active and supportive families. None of this might be true for nonresponders. Furthermore, these patients had survived longer than all fast progressors, with mean survival time of 2 years or less.^4^ Thus, the presented conclusions are not valid for every patient with ALS. We lack information on the percentage of those who preserve well-being in LIS compared to those who do not. Yet, there is a subgroup of individuals with good preconditions who find their way to emotionally cope with the disease although it was not their primary choice to continue life with invasive techniques. Therefore, it is not true that only those occasional patients who initially favor invasive life-sustaining treatments are those who learn to live their life with invasive techniques. The reported 19 patients represent about 18% of all patients in LIS registered in the patient organization in Poland who have received invasive ventilation (12 in an emergency situation) with no means to discontinue and no other medical circumstances leading to death. The conclusion that a life in LIS is a life in misery in general as many healthy people anticipate^7^ is not necessarily true, as these tentative data from a highly selected sample show. We are unaware of psychosocial adaptation of the nonresponders within this study, so the presented data might not represent them. Longitudinal studies with patients on incident mechanical ventilation are needed to further analyze psychosocial adaptation in LIS in general. Finally, making decisions in ALS is not simply a matter of accepting invasive measures or not, and the presented data are unable to fully describe the highly complex situation of living with ALS. Yet the reported data from a Polish sample are representative for patients in LIS in many European countries with respect to coverage of costs (all medical costs are covered by health insurance) and a general right to take life-sustaining measures such as invasive ventilation and PEG.^32^

Overall, there are subgroups of patients who end up in a LIS and who develop a positive mental attitude despite the devastating physical condition. Therapeutic devices that significantly extend life are favored by these patients and the wish for hastened death is low.

## Glossary

ACSA: Anamnestic Comparative Self-Assessment
ADI-12: ALS Depression Inventory–12 items
ALS: amyotrophic lateral sclerosis
ALSFRS-R: ALS Functional Rating Scale–revised version
LIS: locked-in state
NIV: noninvasive ventilation
PEG: percutaneous endoscopic gastrostomy
QoL: quality of life
SAHD: schedule of attitudes toward hastened death
SEIQoL-DW: Schedule for the Evaluation of Individual Quality of Life-Direct Weighting

## References

[1] Plum F, Posner JB. The Diagnosis of Stupor and Coma. Philadelphia: FA Davis; 1966.

[2] Bourke SC, Tomlinson M, Williams TL, et al. Effects of non-invasive ventilation on survival and quality of life in patients with amyotrophic lateral sclerosis: a randomised controlled trial. Lancet Neurol 2006;5:140–147. 16426990 10.1016/S1474-4422(05)70326-4

[3] Gregory S, Siderowf A, Golaszewski AL, McCluskey L. Gastrostomy insertion in ALS patients with low vital capacity: respiratory support and survival. Neurology 2002;58:485–487. 11839859 10.1212/wnl.58.3.485

[4] Lulé D, Ehlich B, Lang D, et al. Quality of life in fatal disease: the flawed judgement of the social environment. J Neurol 2013;260:2836–2843. 23989341 10.1007/s00415-013-7068-y

[5] Heritier Barras AC, Adler D, Iancu Ferfoglia R, et al. Is tracheostomy still an option in amyotrophic lateral sclerosis? Reflections of a multidisciplinary work group. Swiss Med Wkly 2013;143:w13830. 23925784 10.4414/smw.2013.13830

[6] Felgoise SH, Chakraborty BH, Bond E, et al. Psychological morbidity in ALS: the importance of psychological assessment beyond depression alone. Amyotroph Lateral Scler 2010;11:351. 20235756 10.3109/17482961003667630

[7] Lulé D, Häcker S, Ludolph A, et al. Depression and quality of life in patients with amyotrophic lateral sclerosis. Dtsch Arztebl Int 2008;105:397–403. 19626161 10.3238/arztebl.2008.0397 PMC2696844

[8] Lulé D, Nonnenmacher S, Sorg S, et al. Live and let die: existential decision processes in a fatal disease. J Neurol 2014;261:518–525. 24413639 10.1007/s00415-013-7229-z

[9] Roos E, Mariosa D, Ingre C, et al. Depression in amyotrophic lateral sclerosis. Neurology 2016;86:2271–2277. 27164661 10.1212/WNL.0000000000002671 PMC4909561

[10] Miller GA, Galanter E, Pribram KA. Plans and the Structure of Behavior. New York: Holt, Rhinehart, & Winston; 1960.

[11] Linse K, Rüger W, Joos M, Schmitz-Peiffer H, Storch A, Hermann A. Eye-tracking-based assessment suggests preserved well-being in locked-in patients. Ann Neurol 2017;81:310–315. 28074605 10.1002/ana.24871

[12] Lulé D, Zickler C, Häcker S, et al. Life can be worth living in locked-in syndrome. Prog Brain Res 2009;177:339–351. 19818912 10.1016/S0079-6123(09)17723-3

[13] Laureys S, Pellas F, Van Eeckhout P, et al. The locked-in syndrome: what is it like to be conscious but paralyzed and voiceless? Prog Brain Res 2005;150:495–511. 16186044 10.1016/S0079-6123(05)50034-7

[14] Brooks BR, Miller RG, Swash M, et al. El Escorial revisited: revised criteria for the diagnosis of amyotrophic lateral sclerosis. Amyotroph Lateral Scler Other Mot Neuron Disord 2000;1:293–299. 10.1080/146608200300079536 11464847

[15] Rascovsky K, Hodges JR, Knopman D, et al. Sensitivity of revised diagnostic criteria for the behavioural variant of frontotemporal dementia. Brain 2011;134:2456–2477. 21810890 10.1093/brain/awr179 PMC3170532

[16] Gorno-Tempini ML, Hillis AE, Weintraub S, et al. Classification of primary progressive aphasia and its variants. Neurology 2011;76:1006–1014. 21325651 10.1212/WNL.0b013e31821103e6 PMC3059138

[17] Cedarbaum JM, Stambler N, Malta E, et al. The ALSFRS-R: a revised ALS functional rating scale that incorporates assessments of respiratory function: BDNF ALS Study Group (Phase III). J Neurol Sci 1999;169:13–21. 10540002 10.1016/s0022-510x(99)00210-5

[18] Böhm S, Aho-Özhan HE, Keller J, et al. Medical decisions are independent of cognitive impairment in amyotrophic lateral sclerosis. Neurology 2016;87:1737–1738. 27664982 10.1212/WNL.0000000000003232

[19] Bernheim JL. How to get serious answers to the serious question: “How have you been?” Subjective quality of life (QOL) as an individual experiential emergent construct. Bioethics 1999;13:272–287. 11657238 10.1111/1467-8519.00156

[20] O'Boyle C, McGee H, Hickey A, et al. The Schedule for the Evaluation of Individual Quality of Life (SEIQoL). Administration Manual. Psychology Reports; 1993. Available at: epubs.rcsi.ie/psycholrep/39. Accessed August 4, 2008.

[21] Hammer EM, Hacker S, Hautzinger M, et al. Validity of the ALS Depression Inventory (ADI-12): a new screening instrument for depressive disorders in patients with amyotrophic lateral sclerosis. J Affect Disord 2008;109:213–219. 18262283 10.1016/j.jad.2007.11.012

[22] Rosenfeld B, Breitbart W, Stein K, et al. Measuring desire for death among the medically ill: the schedule of attitudes toward hastened death. Am J Psychiatry 1999;156:94–100. 9892303 10.1176/ajp.156.1.94

[23] Andersen PM, Kuzma-Kozakiewicz M, Keller J, et al. Therapeutic decisions in ALS patients: cross-cultural differences and clinical implications. J Neurol 2018;265:1600–1606. 29728768 10.1007/s00415-018-8861-4

[24] Lee JN, Rigby SA, Burchardt F, et al. Quality of life issues in motor neuron disease: the development and validation of a coping strategies questionnaire, the MND Coping Scale. J Neurol Sci 2001;191:79–85. 11676996 10.1016/s0022-510x(01)00619-0

[25] Herschbach P. The well-being paradox in quality-of-life research: on what does our sense of well-being depend? Psychother Psych Med 2002;52:141–150. 10.1055/s-2002-24953 11941521

[26] Bernard M, Strasser F, Gamondi C, et al; SMILE consortium team. Relationship between spirituality, meaning in life, psychological distress, wish for hastened death, and their influence on quality of life in palliative care patients. J Pain Symptom Manage 2017;54:514–522. 28716616 10.1016/j.jpainsymman.2017.07.019

[27] Lulé D, Pauli S, Altintas E, et al. Emotional adjustment in amyotrophic lateral sclerosis (ALS). J Neurol 2012;259:334–341. 21808983 10.1007/s00415-011-6191-x

[28] Matuz T, Birbaumer N, Hautzinger M, Kübler A. Coping with amyotrophic lateral sclerosis: an integrative view. J Neurol Neurosurg Psychiatry 2010;81:893–898. 20587497 10.1136/jnnp.2009.201285

[29] Narayanaswami P, Bertorini TE, Pourmand R, Horner LH. Long-term tracheostomy ventilation in neuromuscular diseases: patient acceptance and quality of life. Neurorehabil Neural Repair 2000;14:135–139. 15470824 10.1177/154596830001400206

[30] Aho-Özhan HE, Böhm S, Keller J, et al. Experience matters: neurologists' perspectives on ALS patients' well-being. J Neurol 2017;264:639–646. 28120043 10.1007/s00415-016-8382-y

[31] Burke T, Galvin M, Pinto-Grau M, et al. Caregivers of patients with amyotrophic lateral sclerosis: investigating quality of life, caregiver burden, service engagement, and patient survival. J Neurol 2017;264:898–904. 28280986 10.1007/s00415-017-8448-5

[32] Weber C, Fijalkowska B, Ciecwierska K, et al. Existential decision-making in a fatal progressive disease: how much do legal and medical frameworks matter? BMC Palliat Care 2017;16:80. 29284475 10.1186/s12904-017-0252-6 PMC5745921

